# End-stage renal disease treated in Provence-Alpes Côte d’Azur: 12-years follow-up and forecast to the year 2030

**DOI:** 10.1186/s12882-018-0929-y

**Published:** 2018-06-15

**Authors:** Anne-Claire Durand, Elisabeth Jouve, Jean-Christophe Delarozière, Mohamed Boucekine, Ghizlane Izaaryene, Adeline Crémades, Franck Mazoué, Bénédicte Devictor, Asmatullah Kakar, Roland Sambuc, Philippe Brunet, Stéphanie Gentile

**Affiliations:** 1 Aix-Marseille University, EA 3279, Public Health, Chronic Diseases and Quality of Life research Unit, 27 Boulevard Jean Moulin, 13385 Marseille cedex 5, France; 2 Cellule d’appui épidémiologique, registre REIN Provence-Alpes Côtes d’Azur, Hôpital de La Conception, Assistance Publique - Hôpitaux de Marseille, Marseille, France; 3 Service d’Evaluation Médicale, Hôpital de La Conception, Assistance Publique - Hôpitaux de Marseille, Marseille, France; 4 Department of nephrology and renal transplantation, Hôpital de La Conception, Assistance Publique - Hôpitaux de Marseille, Marseille, France

**Keywords:** End-stage renal disease, Dialysis, Renal transplant, Time trends, Forecast, Preventive measures

## Abstract

**Background:**

This study describes the time trend of renal replacement therapy for end-stage renal disease (ESRD) in the Provence-Alpes Côte d’Azur region (PACA) between 2004 and 2015, and forecasts up to 2030.

**Methods:**

A longitudinal study was conducted on all ESRD patients treated in PACA and recorded in the French Renal Epidemiology and Information Network (REIN) during this period. Time trends and forecasts to 2030 were analyzed using Poisson regression models.

**Results:**

Since 2004, the number of new patients has steadily increased by 3.4% per year (95% CI, 2.8–3.9, *p* < 0.001) and the number of patients receiving RRT has increased by 3.7% per year (RR 1.037, 95% CI: 1.034–1.039, *p* < 0.001). If these trends continue, the PACA region will be face with 7371 patients on dialysis and 3891 with a functional renal transplant who will need to be managed in 2030. The two most significant growth rates were the percentage of obese people (RR 1.140, 95% CI: 1.131–1.149, *p* < 0.001) and those with diabetes (RR 1.070, 95% CI; 1.064–1.075, *p* < 0.001).

**Conclusion:**

This study highlights the increase in the number of ESRD patients over 12 years, with no prospect of stabilization. These findings allow us to anticipate the quality and quantity of care offered and to propose more preventive measures to combat obesity and diabetes.

## Background

The management of end-stage renal disease (ESRD) continues to be one of the main public health priorities in France due to the increasing number of patients requiring renal replacement therapy (RRT) [1]. On December 31, 2015, 82,295 patients were receiving a RRT: 54% were on dialysis and 46% were living with a functional renal transplant [2]. The overall crude prevalence was 1232 per million inhabitants. The management of ESRD is still a major economic challenge [1]. In France, health insurance had estimated its cost to more than 4 billion euros in 2007 on the basis of 61,000 patients treated for ESRD and 5 billion in 2025, simply because of the ageing of the population, without taking into account the increase in prevalence [1, 3, 4].

In France, two registries developed and managed by the French Biomedicine Agency include and follow ESRD patients: the French Renal Epidemiology and Information Network (REIN) [5, 6] created in 2001 and specific to dialysis patients, and the CRISTAL registry specific to transplant patients [7, 8] collecting social and medical data for all patients receiving an organ transplant.

REIN is a network of nephrologists, epidemiologists, patients and public health representatives that is coordinated regionally and nationally. REIN is a tool to support both public health decision-making, evaluation and research related to RRT for ESRD [5, 6], its data are particularly used in health planning to adjust dialysis supply and demand [9].

In 2004, the Provence-Alpes-Côte d’Azur (PACA) region was the seventh region among the 27 French regions that integrated the REIN registry [10, 11]. This region is located in the south-east of France and represents 7.5% of the French population. The main epidemiological characteristic of PACA is the high proportion of elderly people (19% are aged 65 and over compared to 17% for France) [12].

In the PACA region, there are 81 dialysis centers including 2 pediatrics centers and 2 transplant units [11]. Among these dialysis facilities, we distinguish by the techniques (haemodialysis (HD) and peritoneal dialysis (PD)), dialysis sites (in care center or at home), and types of dialysis centers (in-center-HD, HD in medical satellite unit, HD in self-care unit). There is therefore a gradation of the supply of care with these treatment possibilities [1].

Most of reports provide age-standardized incidence and prevalence rates to describe the epidemiological data of REIN. This presentation is reliable when comparing data between countries and/or regions. However, it is not relevant enough to estimate the number of dialysis stations. Epidemiological data from the REIN registry therefore allow prospective reflection to assess the demand and types of care to be deployed in health planning for the coming years.

The aim of this article is to describe time trends of ESRD treated with RRT (dialysis and renal transplantation) in the PACA region over the last 12 years (2004–2015) based on data collected in the REIN registry, and to forecast these data to 2030.

## Methods

### Type of study

A longitudinal study was carried out on the yearly aggregate data of all patients with RRT, either dialysis or renal transplantation, in the PACA region.

### Data sources

Data on dialysis patients come from the French REIN registry [5, 6]. The design of the REIN registry with its principles of organization and quality control has been described in detail by Couchoud et al. [6]. Data on transplanted patients come from the CRISTAL registry [7, 8].

### Study population and period of analysis

We include all patients, both children and adults, with ESRD undergoing RRT (dialysis or renal transplantation) between 2004 and 2015 treated in PACA, regardless of their place of residence. We excluded patients treated outside the region even if they lived in PACA, and patients diagnosed with acute renal failure (i.e. patients who recovered all or part of their renal function within 45 days, or who were considered by experts to have had acute renal failure for less than 45 days before to death).

We focused on two categories of ESRD patients. The first category included new patients defined as all patients who started their first RRT (dialysis or pre-emptive renal transplantation) in a given year. The second category included patients receiving RRT, on dialysis or living with a renal transplant on December 31 of a given year. We used aggregate data to explore trends over time.

### Data collected

For the REIN registry, data are collected by nephrologists or by clinical research assistants specific to REIN from patient files and recorded in the computer system of REIN (Diadem) [5]. Quality control and data updating each year at the anniversary date of the first dialysis are performed by clinical research assistants. Completeness and accuracy are systematically checked for the items deemed essential, i.e. identification (place of residence), demographics, primary renal disease, date at RRT initiation, comorbid conditions, treatment modalities, and major events including renal transplantation, changes in dialysis site, changes in type of dialysis, transient recovery of renal function and death [5, 6].

For the CRISTAL registry, health professionals and clinical research assistants specific to CRISTAL continuously collect and update the following information: administrative, medical, biological and all events (including transplants performed, functional transplants and patients on the transplant waiting list, regardless of organ and type of donor) [7, 8].

### Analyzed data

First, analyzed data were the annual number of new patients who started their first RRT (initial dialysis and pre-emptive renal transplantation) in a given year, the annual number of patients treated by dialysis or living with a renal transplant on December 31 of a given year, and the annual number of renal transplants performed in PACA (including pre-emptive renal transplants and the type of donor (cadaveric or living donor)).

Secondly, we studied three categories of yearly aggregate variables for new patients (dialysis and pre-emptive renal transplantation) and for patients on dialysis or living with a renal transplant. The first category included demographic data: age, gender, and region of residence (grouped as residing in PACA and residing outside the region).

The second category covered clinical data. Three comorbidities were considered: diabetes, at least one cardiovascular disease (combination of congestive heart failure, myocardial infarction, coronary vascular disease, coronary artery bypass surgery, angioplasty or abnormal angiography, peripheral vascular disease, cerebrovascular disease, and dysrhythmia), and active malignancy. Body mass index (BMI) was also included, calculated by weight ((kg)/square height (m)) and presented in the following classes: underweight (< 18.5), normal (18.5–24.9), overweight (25.0–29.9) and obese (≥30). All comorbidities are collected from the information contained in the patient file completed by the nephrologist who manages the patient.

The third category included data related to dialysis techniques (grouped in HD and PD) and treatment modalities (grouped in in-center-HD, HD in medical satellite unit, HD in self-care unit and home (DP or HD)).

There were no missing data on age, gender, residence, and dialysis treatment (mandatory data).

### Data analysis

SPSS Statistics 20. software was used for all statistical analyses. Qualitative data (i.e. gender, comorbidities, BMI categories, treatment modalities, living with a functional renal transplant) were presented in terms of number of patients and percentage. Quantitative data (age, number of comorbidities) were expressed as mean ± standard deviation (SD). All these data are yearly aggregated data.

To examine time trends of ESRD patients over the period 2004 to 2015, and to forecast the annual number of new patients, the annual number of patients receiving RRT, and the annual number of renal transplants performed to 2030, we used Poisson regression models with the observed number of ESRD patients as the outcome variable and the calendar year as the predictor. This method allows the estimation of time trends across individual calendar years to obtain the average annual percentage change (AAPC), assuming that the rate of change is constant to the previous year [13]. The Poisson regression procedure corresponds to a model of the following form:

$$ Log\left({y}_n\right)={\beta}_0+{\beta}_1\times Time $$

where y_n_ is the number of ESRD patients per year, Log is the natural log, β_0_ the intercept, β_1_ the trend and Time the year- year is given as 0, 1, 2,…,11 (year 0 is 2004, year 1 is 2005 and so on to 2015).

The AAPC was calculated using the following formula:

$$ AAPC=\left( RR-1\right)\times 100, $$

where RR=$$ {e}^{\beta_1} $$

The significance of the trend was determined by the Wald Chi-Square test statistic. The likelihood ratio test was examined for model fit. *P*-values < 0.05 indicate that the model is significantly better than the model without a “Time” predictor. This model was then used to predict the number of ESRD patients to 2030.

## Results

### Trends of initial RRT (dialysis or with a pre-emptive renal transplant) since 2004

Between January 1, 2004 and December 31, 2015, a total of 10,055 new patients started their first RRT in the PACA region. Of these, 9822 patients started a treatment by dialysis (97.7%) and 233 received a pre-emptive renal transplant (2.3%) (Table 1). Since 2004, the number of new patients has steadily increased by 3.4% per year (Rate ratio (RR) 1.034, 95% CI: 1.028–1.039) (Table 2).

**Table 1:** New patients starting their first renal replacement therapy (dialysis or with a pre-emptive kidney transplant) in the PACA region from 2014 to 2015

	2004	2005	2006	2007	2008	2009	2010	2011	2012	2013	2014	2015
All new patients starting RRT	664	739	737	762	808	837	832	912	889	913	951	1011
Pre-emptive transplant, *n* (%)	7 (1.1)	5 (0.7)	6 (0.8)	15 (2.0)	24 (3.0)	11 (1.3)	21 (2.5)	22 (2.4)	21 (2.4)	35 (3.8)	37 (3.9)	29 (2.9)
Initial dialysis, *n* (%)	657 (98.9)	734 (99.3)	731 (99.2)	747 (98.0)	784 (97.0)	826 (98.7)	811 (97.5)	890 (97.6)	868 (97.6)	878 (96.2)	914 (96.1)	982 (97.1)
Characteristics of the new patients starting their first dialysis^a^												
Age Mean ± SD^b^	68.5 ± 15.7	67.9 ± 15.5	68.4 ± 15.5	68.1 ± 15.8	70.0 ± 14.7	68.3 ± 16.8	70.6 ± 15.1	70.7 ± 15.3	71.1 ± 15.4	70.3 ± 14.6	70.9 ± 14.8	70.1 ± 16.5
< 20 years, *n* (%)	9 (1.4)	4 (0.5)	7 (1.0)	9 (1.2)	7 (0.9)	15 (1.8)	10 (1.2)	10 (1.1)	6 (0.7)	6 (0.7)	2 (0.2)	14 (1.4)
≥ 80 years, *n* (%)	160 (24.4)	154 (21.0)	167 (22.8)	172 (23.0)	214 (27.3)	241 (29.2)	246 (30.3)	278 (31.2)	287 (33.1)	255 (29.0)	295 (32.3)	315 (32.1)
Gender: Male, *n* (%)	416 (63.3)	448 (61.0)	460 (62.9)	484 (64.8)	474 (60.5)	554 (67.1)	527 (65.0)	566 (63.7)	587 (67.6)	586 (66.7)	589 (64.4)	644 (65.6)
Residing outside the region PACA, *n* (%)	15 (2.3)	20 (2.7)	15 (2.1)	13 (1.7)	18 (2.3)	26 (3.1)	16 (2.0)	22 (2.5)	28 (3.2)	13 (1.5)	10 (1.1)	15 (1.5)
Comorbidities												
*MD* ^*c*^ *=4.2% (13.4–0.4)*												
None	151 (23.0)	167 (22.8)	178 (24.4)	203 (27.2)	190 (24.2)	244 (29.5)	200 (24.7)	191 (21.5)	206 (23.7)	202 (23.0)	171 (18.7)	229 (23.3)
Mean number ± SD^a^	2.2 ± 1.3	2.1 ± 1.3	2.2 ± 1.4	2.1 ± 1.3	2.1 ± 1.2	2.3 ± 1.4	2.2 ± 1.3	2.5 ± 1.4	2.4 ± 1.4	2.5 ± 1.6	2.4 ± 1.5	2.4 ± 1.6
Diabetes, *n* (%)	212	253	252	243	302	290	306	356	345	368	389	400
*MD = 4.4% (10.6–0.2)*	(37.2)	(39.8)	(35.9)	(36.1)	(40.3)	(35.3)	(38.1)	(40.4)	(40.0)	(42.2)	(42.9)	(41.0)
At least one CVD^d^	307	319	371	335	388	418	440	525	497	490	546	546
*MD = 5.3% (14.3–1.1)*	(54.5)	(50.6)	(52.9)	(50.5)	(52.2)	(51.7)	(56.0)	(60.1)	(57.9)	(56.5)	(60.5)	(56.9)
Cancer	37	60	68	53	65	59	77	86	106	102	100	112
*MD = 4.9% (10.9–0.6)*	(6.6)	(9.5)	(9.7)	(8.0)	(8.7)	(7.4)	(9.9)	(9.9)	(12.5)	(11.8)	(11.1)	(11.7)
BMI^e^, *n* (%)												
*MD = 22.6% (57.4–2.7)*												
Underweight (< 18.5)	38 (7.9)	39 (7.2)	29 (5.6)	37 (7.3)	44 (7.7)	39 (5.9)	46 (6.7)	58 (7.4)	41 (5.4)	37 (4.6)	46 (5.5)	38 (4.3)
Normal (18.5–24.9)	239 (49.7)	273 (50.3)	231 (44.6)	259 (51.2)	272 (47.5)	315 (47.7)	336 (48.8)	363 (46.1)	321 (41.9)	344 (42.4)	330 (39.1)	366 (41.7)
Overweight (25.0–29.9)	149 (31.0)	155 (28.5)	175 (33.8)	151 (29.8)	177 (30.9)	203 (30.7)	182 (26.5)	230 (29.2)	257 (33.6)	256 (31.5)	259 (30.7)	297 (33.8)
Obese (≥ 30)	55 (11.4)	76 (14.0)	83 (16.0)	59 (11.7)	80 (14.0)	104 (15.7)	124 (18.0)	137 (17.4)	147 (19.2)	175 (21.6)	209 (24.8)	177 (20.2)
Initial dialysis techniques and modalities, n (%)												
DP^f^	49 (7.5)	58 (7.9)	49 (6.7)	46 (6.2)	32 (4.1)	50 (6.1)	49 (6.0)	59 (6.6)	65 (7.5)	71 (8.1)	71 (7.8)	81 (8.2)
HD^g^	608 (92.5)	676 (92.1)	682 (93.3)	701 (93.8)	752 (95.9)	776 (93.9)	762 (94.0)	831 (93.4)	803 (92.5)	807 (91.9)	843 (92.2)	901 (91.8)
In-center HD	543 (89.3)	608 (89.9)	622 (91.2)	622 (88.7)	676 (89.9)	691 (89.0)	694 (91.1)	729 (87.7)	745 (92.8)	729 (90.3)	777 (92.2)	816 (90.6)
HD in medical satellite unit	–	1 (0.1)	4 (0.6)	10 (1.4)	11 (1.5)	15 (1.9)	18 (2.4)	32 (3.9)	25 (3.1)	38 (4.7)	38 (4.5)	47 (50.2)
HD in self-care unit	54 (8.9)	54 (8.0)	40 (5.9)	51 (7.3)	43 (5.7)	41 (5.3)	33 (4.3)	33 (4.0)	13 (1.6)	22 (2.7)	22 (2.6)	28 (3.1)
HD at home	11 (1.8)	13 (1.9)	15 (2.2)	18 (2.6)	22 (2.9)	29 (3.7)	17 (2.2)	37 (4.5)	20 (2.5)	18 (2.2)	6 (0.7)	10 (1.1)

^a^There were no missing data for age, gender, residence, and treatment by dialysis (mandatory data)

^b^*SD* Standard Deviation, ^c^*MD* Mean percentage of missing data (Minimum-Maximum), ^d^*CVD* cardiovascular disease (heart failure, coronary heart disease, history of myocardial infarction, aortic aneurysm (as of 2008), dysrhytmia, peripheral vascular disease, cerebrovascular accident or transient ischemic attack), ^e^*BMI* body mass index calculated only for patients aged 18 years and more (kg/m^2^), ^f^*PD* peritoneal dialysis, ^g^*HD* haemodialysis

**Table 2:** Regression analysis of observed number of ESRD patients during the period 2004–2015 in the PACA region

	β (95% CI)	Exp(β) (95% CI)	*p*
New patients starting RRT	0.033 (0.027–0.039)	1.034 (1.028–1.039)	< 0.001
Pre-emptive transplant	0.145 (0.105–0.185)	1.156 (1.111–1.203)	< 0.001
New patients starting their first dialysis	0.031 (0.025–0.036)	1.031 (1.025–1.037)	< 0.001
Age: 80 years and more	0.066 (0.056–0.077)	1.069 (1.057–1.080)	< 0.001
None co-morbidity	0.019 (0.007–0.030)	1.019 (1.007–1.031)	0.002
At least one cardiovascular disease	0.055 (0.047–0.063)	1.057 (1.048–1.065)	< 0.001
Diabetes	0.054 (0.045–0.064)	1.056 (1.046–1.066)	< 0.001
Cancer	0.082 (0.062–0.101)	1.085 (1.064–1.106)	< 0.001
Underweight (< 18.5)	0.016 (− 0.010–0.042)	1.016 (0.990–1.043)	0.220
Normal BMI (18.5–24.9)	0.038 (0.029–0.048)	1.039 (1.029–1.049)	< 0.001
Overweight (25.0–29.9)	0.065 (0.053–0.076)	1.067 (1.055–1.079)	< 0.001
Obese (≥ 30)	0.116 (0.100–0.132)	1.123 (1.105–1.141)	< 0.001
DP	0.050 (0.028–0.071)	1.051 (1.028–1.074)	< 0.001
HD	0.029 (0.023–0.035)	1.030 (1.023–1.036)	< 0.001
In-center HD	0.031 (0.025–0.037)	1.031 (1.025–1.038)	< 0.001
HD in medical satellite unit	0.245 (0.201–0.290)	1.278 (1.222–1.336)	< 0.001
HD in self-care unit	− 0.092 (−0.120 − −0.063)	0.913 (0.887–0.939)	< 0.001
Renal transplant performed	0.050 (0.039–0.062)	1.052 (1.039–1.064)	< 0.001
Living donor	0.190 (0.140–0.241)	1.210 (1.150–1.272)	< 0.001
Deceased donor	0.041 (0.029–0.053)	1.042 (1.029–1.055)	< 0.001
Patients receiving RRT	0.036 (0.034–0.038)	1.037 (1.034–1.039)	< 0.001
Patients with a functional renal transplant	0.055 (0.051–0.059)	1.057 (1.053–1.061)	< 0.001
Patints on dialysis	0.026 (0.023–0.029)	1.026 (1.024–1.029)	< 0.001
Age: 80 years and more	0.105 (0.099–0.111)	1.111 (1.104–1.118)	< 0.001
None co-morbidities	0.006 (0.001–0.012)	1.006 (1.001–1.012)	0.027
Diabetes	0.067 (0.062–0.072)	1.070 (1.064–1.075)	< 0.001
Cancer	0.105 (0.094–0.116)	1.111 (1.099–1.123)	< 0.001
At least one CVD	0.059 (0.055–0.063)	1.061 (1.057–1.065)	< 0.001
Underweight (< 18.5)	0.053 (0.041–0.066)	1.055 (1.041–1.069)	< 0.001
Normal BMI (18.5–24.9)	0.076 (0.071–0.081)	1.079 (1.074–1.084)	< 0.001
Overweight (25.0–29.9)	0.097 (0.091–0.103)	1.102 (1.095–1.108)	< 0.001
Obese (≥ 30)	0.131 (0.123–0.139)	1.140 (1.131–1.149)	< 0.001
DP	0.027 (0.014–0.040)	1.027 (1.014–1.041)	< 0.001
HD	0.027 (0.024–0.030)	1.027 (1.024–1.030)	< 0.001
In-center HD	0.019 (0.015–0.022)	1.019 (1.015–1.022)	< 0.001
HD in medical satellite unit	0.311 (0.299–0.324)	1.365 (1.348–1.382)	< 0.001
HD in self-care unit	−0.035 (−0.041 − −0.028)	0.966 (0.960–0.972)	< 0.001
HD at home	−0.159 (−0.182 − −0.136)	0.853 (0.834–0.873)	< 0.001

Among all the patients who started dialysis in PACA, the number of patients residing outside our region remained low, varying between 1.1 and 3.1% depending on the year. These patients lived in the two border regions: Rhône-Alpes and Languedoc-Roussillon. The percentage of children and adolescents under 20 years of age was very low, ranging from 0.2% to a maximum of 1.8%, depending on the year (Table 1).

Since 2004, the number of ESRD patients who started dialysis has steadily increased by an average of 3.1% per year (Table 2): there were 657 in 2004 and 975 in 2015 (RR 1.031, 95% CI: 1.025–1.037, *p* < 0.001). The annual number of pre-emptive renal transplants increased slightly (RR 1.156, 95% CI: 1.111–1.203, *p* < 0.001).

### Characteristics of new patients starting a treatment by dialysis, PACA, 2004–2015

Table 1 presents the baseline characteristics of patients starting their first dialysis since 2004. It is marked by an increase in the mean age at first dialysis. Between 2004 and 2015, the mean age increased by 1.6 years. This increase was most important among patients over 80 years of age, with an average 6.9% per year (95% CI: 5.7–8.0, *p* < 0.001) (Table 2). The proportion of new patients with comorbidities also increased by an average of 5.6% per year (Tables 1 and 2). Indeed, the percentage of obese patients increased by almost 10 points (RR 1.123, 95% CI: 1.105–1.141, *p* < 0.001), the percentage of patients with cancer by 5 points (RR 1.085, 95% CI: 1.064–1.106, *p* < 0.001), and the percentage of diabetic patients by 3.8 points (RR 1.056, 95% CI: 1.046–1.066, *p* < 0.001).

The percentage of patients starting with PD remained low between 2004 and 2015, and has remained stable over the past 3 years: about 8%. Among HD patients, more than 88% of them started their initial treatment in-center HD (Table 1).

### Number of ESRD patients treated by dialysis or living with a renal transplant in the PACA region at the 31st December between 2004 and 2015

Since 2004, the number of patients receiving RRT has increased by 3.7% per year (RR 1.037, 95% CI: 1.034–1.039, *p* < 0.001) (Table 2): from 4433 patients on December 31, 2004 to 6475 on December 31, 2015 (Table 3).

**Table 3:** Characteristics of ESRD patients treated by dialysis or living with a renal transplant in the PACA region on December 31 of each year between 2004 and 2015

	2004	2005	2006	2007	2008	2009	2010	2011	2012	2013	2014	2015
ESRD patients treated on December 31												
All ESRD	4433	4542	4716	4908	5081	5312	5517	5729	5920	6101	6357	6475
Living with a renal transplant, *n* (%)	1361 (30.7)	1406 (31.0)	1500 (31.8)	1599 (32.6)	1713 (33.7)	1802 (33.9)	1918 (34.8)	2032 (35.5)	2116 (35.7)	2237 (36.7)	2349 (37.0)	2456 (37.9)
On dialysis, n (%)	3072 (69.3)	3136 (69.0)	3216 (68.2)	3309 (67.4)	3368 (66.3)	3510 (66.1)	3599 (65.2)	3697 (64.5)	3804 (64.3)	3864 (63.3)	4008 (63.0)	4019 (62.1)
Characteristics of patients treated by dialysis on December 31^a^												
Age mean ± SD^b^	64.9 ± 15,4	65.0 ± 15,3	65.6 ± 15,0	66.1 ± 14,9	66.9 ± 14,6	67.1 ± 14,7	67.5 ± 15,0	69,4 ± 15,0	69.3 ± 15,2	69.6 ± 14,9	69.8 ± 14,9	70,1 ± 15,1
< 20 years, %	24 (0.8)	20 (0.6)	11 (0.3)	16 (0.5)	11 (0.3)	13 (0.4)	19 (0.5)	16 (0.4)	20 (0.5)	14 (0.4)	14 (0.3)	24 (0.6)
≥ 80 years, %	453 (14.7)	466 (14.9)	481 (15.0)	531 (16.0)	622 (18.5)	672 (19.1)	754 (21.0)	1008 (27.3)	1053 (27.7)	1087 (28.1)	1181 (29.5)	1254 (31.2)
Gender: male, n (%)	1843 (60,0)	1887 (60,2)	1949 (60,6)	2011 (60,8)	2035 (60,4)	2151 (61,3)	2202 (61,2)	2275 (61,5)	2371 (62,3)	2431 (62,9)	2500 (62,4)	2567 (63.9)
Residing outside the PACA region, n (%)	102 (3.3)	102 (3.3)	88 (2.7)	92 (2.8)	91 (2.7)	101 (2.9)	92 (2.6)	89 (2.4)	95 (2.5)	93 (2.4)	101 (2.5)	100 (2.5)
Comorbidities												
*MD* ^*c*^ *=4.6% (10.4–0.2)*												
None, %	753 (24.5)	791 (25.2)	824 (25.6)	889 (26.9)	899 (26.7)	990 (28.2)	980 (27.2)	821 (22.2)	866 (22.8)	874 (22.6)	836 (20.9)	858 (21,3)
Mean number ± SD	2,3 ± 1,4	2,2 ± 1,3	2,1 ± 1,3	2,1 ± 1,3	2,1 ± 1,3	2,1 ± 1,3	2,2 ± 1,4	2,4 ± 1,4	2,5 ± 1,4	2,5 ± 1,5	2,5 ± 1,5	2,5 ± 1,5
Main comorbidities, *n* (%)												
Diabetes	782	837	918	933	1022	1103	1174	1290	1369	1446	1563	1597
*MD = 4.4% (10.6–0.2)*	(28.3)	(29.8)	(31.3)	(30.7)	(32.2)	(32.8)	(33.5)	(35.2)	(36.1)	(37.5)	(39.1)	(39.0)
At least one CVD^d^	1514	1485	1504	1530	1632	1685	1814	2235	2312	2335	2488	2559
*MD = 4.7% (10.8–0.4)*	(54.8)	(53.1)	(51.3)	(50.6)	(51.6)	(50.3)	(52.2)	(60.9)	(61.1)	(60.6)	(62.4)	(62.7)
Cancer	144	147	163	177	192	199	237	296	322	352	373	408
*MD = 4.9% (10.9–0.6)*	(5.2)	(5.3)	(5.6)	(5.9)	(6.1)	(6.0)	(6.9)	(8.1)	(8.5)	(9.2)	(9.4)	(10.0)
*BMI*^e^ in kg/m^2^, %												
*MD = 22.6% (57.4 in 2004--2.7)*												
Underweight (< 18.5)	104 (8.0)	135 (7.7)	136 (7.1)	139 (6.6)	155 (6.6)	155 (5.8)	169 (5.8)	202 (5.8)	192 (5.4)	193 (5.2)	201 (5.3)	204 (5.2)
Normal (18.5–24.9)	654 (50.5)	867 (49.8)	915 (47.4)	1030 (48.7)	1144 (48.5)	1298 (48.5)	1444 (49.4)	1657 (47.9)	1675 (46.7)	1644 (44.7)	1623 (42.5)	1666 (42.7)
Overweight (25.0–29.9)	385 (29.7)	506 (29.0)	590 (30.6)	645 (30.5)	718 (30.4)	818 (30.6)	851 (29.1)	1045 (30.2)	1117 (31.1)	1148 (31.2)	1224 (32.0)	1251 (32.1)
Obese (≥ 30)	11.7	13.4	14.9	14.2	14.5	15.1	15.8	16.0	16.8	18.8	20.2	20.0
*Current dialysis modalities*, %												
DP^f^	149 (4,9)	158 (5,0)	146 (4,5)	140 (4,2)	120 (3,6)	126 (3,6)	136 (3,8)	152 (4,1)	161 (4,2)	173 (4,5)	187 (4,7)	196 (4,9)
HD^g^	2923 (95,1)	2978 (95,0)	3070 (95,5)	3169 (95,8)	3248 (96,4)	3384 (96,4)	3463 (96,2)	3545 (95,9)	3643 (95,8)	3691 (95,5)	3821 (95,3)	3913 (97.4)
In-center HD	2093 (71,6)	2126 (71,4)	2228 (72,6)	2309 (72,9)	2335 (71,9)	2381 (70,4)	2437 (70,4)	2449 (69,1)	2483 (68,2)	2484 (67,3)	2566 (67,2)	2603 (66,5)
HD in medical satellite unit	1 (0.003)	3 (0,1)	9 (0,3)	39 (1,2)	79 (2,4)	194 (5,7)	314 (9,1)	441 (12,4)	552 (15,2)	625 (16,9)	706 (18,5)	755 (19,3)
HD in self-care unit	672 (23,0)	745 (25,0)	737 (24,0)	752 (23,7)	779 (24,0)	760 (22,5)	673 (19,4)	619 (17,5)	567 (15,6)	539 (14,6)	522 (13,7)	525 (13,4)
HD at home	157 (5,4)	104 (3,5)	96 (3,1)	69 (2,2)	55 (1,7)	49 (1,4)	39 (1,1)	36 (1,0)	41 (1,1)	43 (1,2)	27 (0,7)	30 (0,8)

^a^There were no missing data for age, gender, residence, and treatment by dialysis (mandatory data)

^b^*SD* Standard Deviation, ^c^*MD* Mean percentage of missing data (Minimum-Maximum), ^d^*CVD* cardiovascular disease (heart failure, coronary heart disease, history of myocardial infarction, aortic aneurysm (as of 2008), dysrhytmia, peripheral vascular disease, cerebrovascular accident or transient ischemic attack), ^e^*BMI* body mass index calculated only for patients aged 18 years and more, ^f^*PD* peritoneal dialysis, ^g^*HD* haemodialysis

Approximately 1000 additional patients were counted in each of the two methods of RRT during this period (Tables 2 and 3): 947 additional patients requiring dialysis (RR 1.026, 95% CI: 1.024–1.09, *p* < 0.001), and 1095 additional patients living with a renal transplant (RR 1.057, 95% CI: 1.053–1.061, *p* < 0.001).

The evolution of the characteristics of dialysis patients (Tables 3) is marked by a significant increase in the mean age of 5.2 years (from 64.9 years in 2004 to 70.1 years in 2015) and a higher proportion of patients with comorbidities. As for the characteristics of patients starting their first dialysis, an increase in the proportion of obese patients (RR 1.140, 95% CI: 1.131–1.149, *p* < 0.001) and the proportion of diabetic patients (RR 1.070, 95% CI: 1.064–1.075, *p* < 0.001), with + 8.3 points and + 10.7 points respectively (Tables 2 and 3).

The percentage of patients treated by PD remained low, not exceeding 5%, and stable over time. As of December 31, the main dialysis technique was HD (≥ 95% of all dialyzed patients each year). Among them, the proportion of patients treated by in-center HD increased (RR 0.01.9, 95% CI: 1.015–1.022, *p* < 0.001). Those treated by HD in self-care unit (RR 0.966, 95% CI: 0.960–0.975, *p* < 0.001) have gradually decreased since 2004 in favor of HD in medical satellite unit, which represents a significant proportion of the treatment site since 2006 (RR 1.365, 95% CI: 1.348–1.382, *p* < 0.001) (Tables 2 and 3).

### Number of renal transplants performed in the PACA region: Trends since 2004

**Fig. 1:**
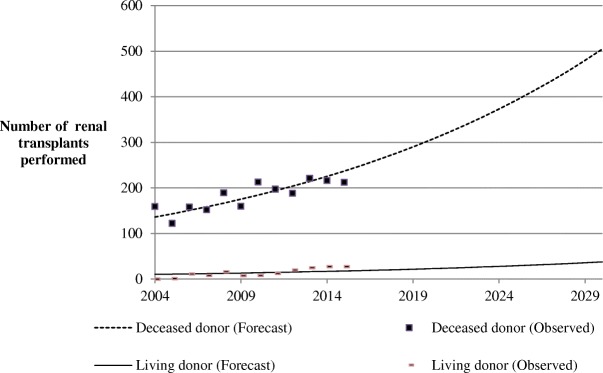
Trends of renal transplants performed in the PACA region from 2004 to 2015 and forecast to the year 2030

Since 2004, the number of renal transplants performed in PACA has gradually increased: 5.2% per year on average (RR 1.052, 95% CI: 1.039–1.064, *p* < 0.001) (Tables 2 and 4). Although transplants are mainly from deceased donors, the proportion of living donors also increased: none in 2004 to 27 in 2015 (Fig. 1).

**Table 4:** Renal transplants performed (including pre-emptive transplant) in the PACA region from 2004 to 2015

	2004	2005	2006	2007	2008	2009	2010	2011	2012	2013	2014	2015
Number of renal transplants performed	159	123	169	160	205	167	221	209	208	246	243	239
Donor type, n (%)												
Deceased donor	159 (100,0)	123 (99,2)	169 (93,5)	160 (95,0)	205 (92,2)	167 (95,8)	221 (96,4)	209 (94,3)	208 (90,4)	246 (89,8)	243 (88,9)	239 (88,7)
Living donor	–	1 (0,8)	11 (6,5)	8 (5,0)	16 (7,8)	7 (4,2)	8 (3,6)	12 (5,7)	20 (9,6)	25 (10,1)	27 (11,1)	27 (11,3)

### Number of ESRD patients undergoing RRT and forecast until 2030

**Fig. 2:**
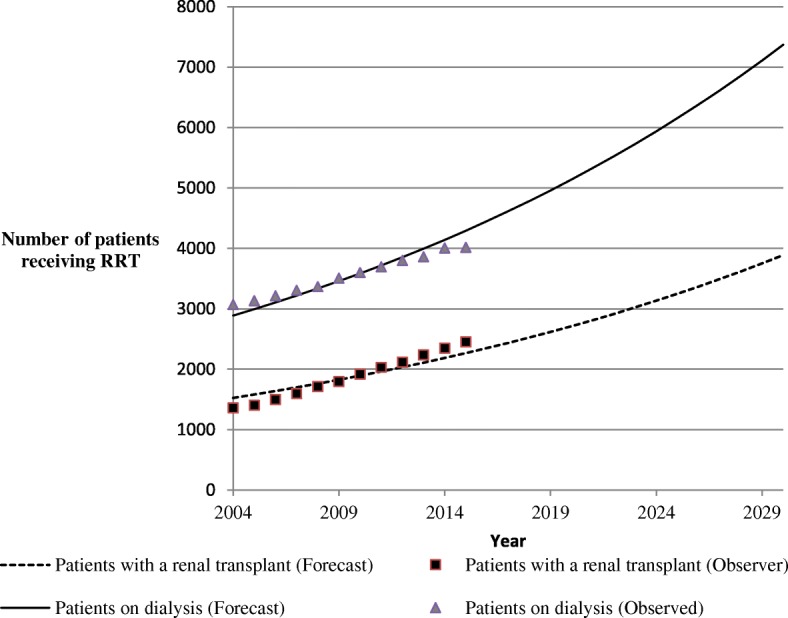
Trends and forecasts of ESRD patients treated by dialysis and living with a renal transplant in the PACA region from 2004 up to 2030

**Fig. 3:**
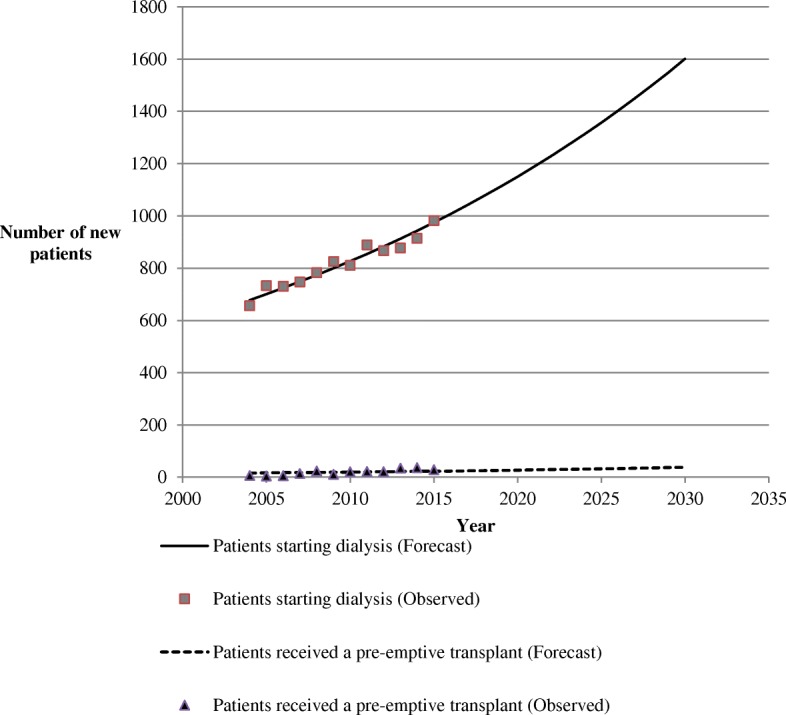
Trends and forecasts of new patients starting their first dialysis and receiving a pre-emptive kidney transplant in the PACA region from 2004 up to 2030

Since 2004, the number of ESRD patients treated by dialysis and living with a renal transplant has increased linearly and continuously (Table 3 and Fig. 2). As shown in Fig. 2, if these trends persist, according to the linear trend model, the PACA region will have approximately 7371 patients on dialysis and 3891 transplant patients to be managed by 2030, representing approximately 3300 additional patients on dialysis compared to in 2015 and 1435 additional patients with functional renal transplant (Fig. 2). For new patients, the PACA region will have approximately 600 additional patients starting their first dialysis who will need to be managed by 2030 (Fig. 3).

## Discussion

Analysis of the data collected in the PACA region between 2004 and 2015 shows that the number of patients with ESRD undergoing RRT is steadily increasing without a tendency to stabilize, as shown by trend lines and forecasts up to 2030. Our results are similar to those of other French regions. The only difference concerns the number of new patients in the PACA region, which is more important than most other French regions (3rd region with the highest number of new patients) [2]. We believe this is due to the large number of elderly patients starting dialysis in our region. Indeed, PACA is the French region with the highest proportion of people aged 75 and over receiving dialysis, behind Ile de France region [2].

This is different from what has been reported by ERA-EDTA registry, the European registry. RRT incidence rates have started to stabilize in Europe since 2008 [14]. France also experienced a period of stability between 2009 and 2011 in its incidence rates. However, incidence rates have started to increase since 2012 due to an increase in the proportion of new patients aged over 85 years [2].

Over the same period, in France as in Europe, there has been an increase in the annual number of renal transplants, with a higher proportion of transplants from living donors [14]. The recent expansion of living donor transplants in France (11% of renal transplants performed in PACA were realized with living donors compared to 16% for the whole of France in 2015 [2]) may change the rate of pre-emptive transplantation in the next few years. Transplantation is the most cost-effective method to provide patients with better quality of life [15, 16]. National discussions are in progress to promote access to transplantation: recently, the ethics committee authorized cross-transplantation [17] and the High Authority of Health issued recommendations on access to the list of transplant recipients waiting for a renal transplantation [18].

Conditions for the management of HD patients have evolved with a smaller proportion of patients treated by in-center HD and self-care unit in favor of a significant increase of HD patients treated in medical satellite unit. It is likely that some patients cared in the medical satellite units were previously treated in the main centers or self-care units and were reoriented as soon as the medical satellite units opened [19, 20]. Indeed, before 2002, many of heavy patients had to be treated in the self-care units because there was no possibility of places available in the center or medical satellite unit [19, 20].

However, in the PACA region, PD was the initial modality of treatment for 8.2% of patients in 2015. The percentage of new patients treated with PD has remained low since 2004. The PACA region is one of the French regions where the proportion of new patients treated with PD remains low (8.2% against 10.3% for France) [2], with no significant progression. The causes are known: for a long time, the lack of sufficient training of nephrologists to PD has been a obstacle on the diffusion of this modality. Today, the nephrology departments of two central university hospitals of PACA, Marseilles and Nice, train young nephrologists in the technique of PD. Thus, it is possible to presume that a much larger number of patients could be treated, as evidenced by the practices of other European countries which support more than 15% of their ESRD patients in PD, i.e. nearly 10 points more than France [21].

The evolution of the characteristics of new patients and those receiving RRT gives us some elements of response with the higher proportion of complex patients: the ageing of patients and the higher number of comorbidities. The other two significant results were the increase in the proportion of obese patients (+ 8.3 points) and the increase in the proportion of diabetic patients (+ 10.7 points).

According to national demographic trends, the proportion of people aged 80 and older increases [22] and it is the main determinant of the increase in the number of patients with ESRD. Moreover, the increase in the incidence of type 2 diabetes in the French population represents an independent risk factor for progression to renal failure [2, 14, 23].

It is impossible to combat ageing, but it is important to continue and reinforce the preventive measures implemented in France since the 1990s, such as the use of renin-angiotensin system inhibitors, better control of blood pressure, moderate restriction of proteins in case of early renal failure, and better management of diabetes. Although international data show the effectiveness of these measures, it has been observed in several countries that the incidence has decreased [14]. In France, two studies showed that the prevention of diabetic nephropathy is not optimal in France [24, 25], in particular the low detection rate of albuminuria and the insufficient use of inhibitors of the renin-angiotensin system in patients with diabetes [24, 25]. Thus, conservative (non-dialytic) management of ESRD in France may not be as developed as in other countries [26, 27].

Finally, there is a need to strengthen current public health measures in the field of combating obesity in children and adults and to make the public and political actors aware of the important consequences that obesity can also have on ESRD.

The magnitude of the study period (12 years) and the completeness of the data collected are the highlights of this study. In addition, we have tested several statistical models, from the simplest to the most complex, the results remain identical: there is a linear increase in the number of ESRD patients since 2004, with no prospect of stabilization until 2030. However, our study also presented some limitations. Our study focused on a single French region that has certain specificities, such as older patients and a lower proportion of PD patients than in other regions.

As for our forecasts, we have limited ourselves to a period of 15 years (until 2030) because we believe that the epidemiological evolution will remain stable. A longer time period would make the model too uncertain. Indeed, whatever the public health measures or technological innovations, the impact on the number of ESRD patients will probably not affect future generations concerned by dialysis by 2030. It is therefore important to provide an intermediate or periodic analysis to document progress. Finally, no information was available for patients with ESRD who were not treated by RRT, which could have biased trends in the RRT incidence.

## Conclusions

The originality of this study is to focus on the number of ESRD patients and not only on incidence rates, which allows us to estimate the number of dialysis places expected in the future. This study highlighted the linear increase in the number of ESRD patients in 12 years, with no prospect of stabilization. These results enable the medical community and health authorities to anticipate the supply of care in qualitative or quantitative terms as well as to be a part of a public health approach aimed at integrating more preventive measures to combat obesity and diabetes in order to expect to stabilize the number of ESRD patients in the future.

## Abbreviations

BMI: Body mass index
CI: Confidential interval
CVD: Cardiovascular disease
ESRD: End-stage renal disease
HD: Haemodialysis
PACA: Provence-Alpes Côte d’Azur Region
PD: Peritoneal dialysis
REIN: Renal epidemiology and information network registry
RR: Rate ratio
RRT: Renal replacement therapy

## References

[1] Haute Autorité de Santé. Evaluation médico-économique des stratégies de prise en charge de l’insuffisance rénale en France - Synthèse et conclusions. Octobre 2014. [Accessed 17th July 2017] Available from: http://www.has-sante.fr/portail/upload/docs/application/pdf/2014-11/synthese_irct_vf.pdf.

[2] Agence de la biomédecine. Rapport annuel REIN 2015. [Accessed 8th January 2018] Available from: https://www.agence-biomedecine.fr/IMG/pdf/rapport_rein_2015.pdf.

[3] Haute Autorité de Santé Evaluation médico-économique des stratégies de prise en charge de l’insuffisance rénale en France - Note de cadrage. Septembre 2010. [Accessed 17th July 2017] Available from: http://www.has-sante.fr/portail/upload/docs/application/pdf/2010-10/note_cadrage_irct_vf.pdf.

[4] Caisse nationale de l'assurance maladie. L'insuffisance rénale chronique : situation actuelle et enjeux - Point d'information. Mars 2010. [Accessed 17th July 2017] Available from: http://www.ameli.fr/fileadmin/user_upload/documents/DP_Insuffisance_renale_chronique.pdf.

[5] Agence de la biomédecine. Le programme REIN. [Accessed 17th July 2017] Available from: http://www.agence-biomedecine.fr/Le-programme-REIN.

[6] Couchoud C, Stengel B, Landais P, Aldigier JC, de CF, Dabot C (2006). The renal epidemiology and information network (REIN). A new regist**r**y for end-stage renal disease in France. Nephrol Dial Transplant.

[7] Strang WN, Tuppin P, Atinault A, Jacquelinet C (2005). The French organ transplant data system. Stud Health Technol Inform.

[8] Agence de la biomédecine. Cristal - L'application internet au service du prélèvement et de la greffe d'organes.[Accessed 17th July 2017] Available from: https://www.agence-biomedecine.fr/IMG/pdf/22559_biom_cristal-2.pdf

[9] Agence Régionale de Santé Provence-Alpes-Côte d'Azur. Schéma Régional d'Organisation de Soins 2012–201. [Accessed 17th July 2017] Available from: https://www.paca.ars.sante.fr/sites/default/files/2017-01/Revision_SROS_PRS_Paca_2012-2016_arspaca_27122013_0.pdf

[10] Agence de la biomédecine (2004). Rapport annuel REIN.

[11] Cellule Epidémiologique REIN Provence Alpes Côtes d'Azur. Rapport REIN 10 ans, 2004-2013. Mars 2015. [Accessed 8th January 2018] Available from:http://www.soc-nephrologie.org/PDF/epart/assoc/ANSEC/REIN-PACA/REIN-PACA-10-ans.pdf

[12] Info Centre des Activités Régionales de Santé (icars). Projet régional de santé 2012–2016. Personnes vieillissantes. Caractéristiques régionale. [Accessed 8th January 2018] Available from: http://www.icarsante-paca.fr/article.php?larub=578&titre=caracteristiques-regionales

[13] Clegg LX, Hankey BF, Tiwari R, Feuer EJ, Edwards BK (2009). Estimating average annual per cent change in trend analysis. Stat Med.

[14] Heaf J (2017). Current trends in European renal epidemiology. Clin Kidney J.

[15] Agence de la biomédecine, Institut de veille sanitaire. Surveillance de la qualité de vie des sujets atteints d’insuffisance rénale chronique terminale. Rapport Qualité de Vie - REIN - Volet greffe 2007. [Accessed 17th July 2017] Available from: http://www.agence-biomedecine.fr/IMG/pdf/rapport_qv_greffe_v1.18_16122009.pdf

[16] Haute Autorité de Santé, Agence de la biomédecine. Evaluation médico-économique des stratégies de prise en charge de l’insuffisance rénale en France. Volet : Analyse des possibilités de développement de la transplantation rénale en France - Recommandation en santé publique Juin 2012. [Accessed 17th July 2017] Available from: http://www.has-sante.fr/portail/upload/docs/application/pdf/2012-09/synthese_irct_volet_greffe_vf.pdf

[17] Agence de la biomédecine (2014). Don croisé. Février.

[18] Haute Autorité de Santé. Transplantation rénale, accès à la liste d’attente nationale (2015). Recommandation de bonne pratique.

[19] Décret n° 2002–1198 du 23 septembre 2002 relatif aux conditions techniques de fonctionnement des établissements de santé qui exercent l'activité de traitement de l'insuffisance rénale chronique par la pratique de l'épuration extrarénale et modifiant le code de la santé publique (troisième partie : Décrets). Journal officiel de la République Française n°224 du 25 septembre 2002.

[20] Circulaire DHOS/SDO n° 228 du 15 mai 2003 relative à l’application des décrets n° 20021197 et 2002–1198 du 23 septembre 2002.

[21] Société Francophone de Néphrologie Dialyse et Transplantation (2016). Rapport sur la dialyse chronique en France en.

[22] Institut national de la statistique et des études économiques. Projections de population à l’horizon 2060. Insee Première N° 1320. p. 2010.

[23] Organisation mondiale de la santé Rapport de la commission pour mettre fin à l'obésité de l'enfant. 2016. [Accessed 17th July 2017] Available from: http://apps.who.int/iris/bitstream/10665/206451/1/9789242510065_fre.pdf?ua=1.

[24] Assogba GF, Couchoud C, Roudier C, Pornet C, Fosse S, Romon I (2012). Prevalence, screening and treatment of chronic kidney disease in people with type 2 diabetes in France: the ENTRED surveys (2001 and 2007). Diabetes Metab.

[25] Vigneau C, Kolko A, Stengel B, Jacquelinet C, Landais P, Rieu P, Bayat S, Couchoud C (2017). REIN registry. Ten-years trends in renal replacement therapy for end-stage renal disease in mainland France: lessons from the French renal epidemiology and information network (REIN) registry. Nephrol Ther.

[26] van de Luijtgaarden MW, Noordzij M, Van BW, Couchoud C, Cancarini G, Bos WJ (2013). Conservative care in Europe–nephrologists’ experience with the decision not to start renal replacement therapy. Nephrol Dial Transplant.

[27] Roderick P, Rayner H, Tonkin-Crine S, Okamoto I, Eyles C, Leydon G, et al. A national study of practice patterns in UK renal units in the use of dialysis and conservative kidney management to treat people aged 75 years and over with chronic kidney failure. Health Serv Deliv Res. 2015; 10.3310/hsdr03120 25855842

